# Prevalence of household falls in long-lived adults and association with extrinsic factors ^1^

**DOI:** 10.1590/1518-8345.1646.2900

**Published:** 2017-10-19

**Authors:** Silviane Galvan Pereira, Claudia Benedita dos Santos, Marlene Doring, Marilene Rodrigues Portella

**Affiliations:** 2 Doctoral student, Escola de Enfermagem de Ribeirão Preto, Universidade de São Paulo, PAHO/WHO Collaborating Centre for Nursing Research Development, Ribeirão Preto, SP, Brazil.; 3 PhD, Associate Professor, Escola de Enfermagem de Ribeirão Preto, Universidade de São Paulo, PAHO/WHO Collaborating Centre for Nursing Research Development, Ribeirão Preto, SP, Brazil.; 4 PhD, Professor, Universidade de Passo Fundo, Passo Fundo, RS, Brazil

**Keywords:** Aged, 80 and Over, Accidental Falls, Risk Factors, Nursing

## Abstract

**Objective:**

to identify the prevalence of falls among older adults and the extrinsic factors associated with them.

**Method:**

population-based cross-sectional study with 350 older adults. A household survey was conducted using a questionnaire addressing socio-demographic, clinical, and environmental characteristics. Data were analyzed using Stata Software V.10. Pearson’s chi-square test and logistic regression analysis were used with stepwise criteria for selection of variables in the model, with measures of effect expressed in Prevalence Ratio. For input into the multiple model, the variables with p ≤ 0.20 were considered. All ethical care regarding research on human beings has been observed and respected.

**Results:**

the prevalence of falls was 46.9%. The extrinsic factors associated with falls were: stairs, uneven floor and pets in the main entrance, lack of anti-slip loose throw rugs and slippery floor in the kitchen, lack of anti-slip loose throw rugs and objects on the floor in the room, lack of grab bars in the shower, lack of grab bars in the toilet and switch away from the bathroom door (p <0.05).

**Conclusion:**

falls are frequent in long-lived adults. The identification of the extrinsic factors associated with the occurrence of this event can help in its prevention.

## Introduction

The aging phenomenon is marked by changes in the demographic structure and in the socioeconomic and health conditions of the population. Its repercussions are felt both by society and by the health system^1^. These transformations demand a more thorough study of this segment and of the problems older adults are exposed to, such as events associated with falls.

The occurrence of falls is considered one of the main external causes of morbidity and mortality among the older adults. A fall is defined as any unexpected contact with the ground by any part of the person’s body, except for the soles of their feet^2^. Events associated with loss of consciousness, blunt cerebrovascular injury, car accident, vigorous recreational activity or violence are often excluded from the definition of falls in older adults^3^.

Falls provoke an important loss of autonomy in older adults^4^ and can cause different consequences, such as mild injuries, fractures and even death^5^. These consequences have an impact on health services, since they increase the use of personal and material resources for medical and nursing care. For this reason, falls are considered the most costly injury among older adults^6^.

Older adults over 80, considered long-lived adults, have alterations in their organism. Among them, sensory and musculoskeletal alterations stand out, since they can cause damages such as an increase in the risk of falls and a reduction in the level of functional independence and, consequently, lead to a decrease in quality of life ^3^. 

In fact, throughout their lives, besides the changes in the biological, psychological and social dimensions, older adults are exposed to several situations that can lead to loss of autonomy and independence, such as the occurrence of falls^7^.

In Brazil, about 30% of older adults suffer falls at least once a year. One in three older adults aged 65 years or older fall one or more times, and half of the older adults who fall suffer a recurrence^8^ ^-^ ^9^. Approximately 2.5% of them require hospitalization; of these, only half survive after one year^9^. The risk of falls almost doubles in individuals over 80 years old. For the long-lived adults, the percentage rises to approximately 50% ^(^ ^10^.

The cumulative effect of changes related to age, diseases and an inadequate environment may predispose people to falls^11^. These events may be associated with risk factors for falls, which may be multifactorial and related to intrinsic and extrinsic factors^12^.

Intrinsic factors are those related to the individual, resulting from the physiological changes brought by aging, the presence of diseases, psychological factors and adverse reactions to medications^9^. Extrinsic factors refer to the behaviors and the activities of older adults and their physical environment, factors that depend on social and environmental circumstances^9^. 

In the review of the specialized literature, a study addressing the interaction of older adults with the environment in relation to falls was found^13^. The findings highlighted the interactions between personal factors and the environment, however, it was not possible to find conclusive answers regarding the relationship between older adults and the environment and the risk of falls. The knowledge of these factors is an important asset for the health care team to establish the necessary foundations for a care system and, consequently, propose measures to prevent future falls.

Regarding the falls suffered by older adults in the community described in the international literature, the greatest interest has been the prevention of falls^14^. On the other hand, most of the studies were conducted in the South and Southeast regions, and the most assessed aspects were: prevalence, incidence, causes and consequences of falls, risk factors and profile of older adults who fell^3^ ^-^ ^4^ ^,^ ^8^. Therefore, studies addressing extrinsic risk factors for falls in older adults are important in order to understand the magnitude and characteristics of this event.

In this sense, a gap in the literature addressing this problem is identified. An extensive review of the literature performed by Spanish-speaking authors revealed there is only a single research addressing the problem of falls in a nursing home^15^, which identified the main extrinsic risk factors as related to architecture, furniture, equipment and processes. 

It should be noted that validated instruments for assessing extrinsic factors or determinants for home falls in the elderly population were not found in the Brazilian literature. 

This study is justified by the increase in the proportion of older adults and falls, which makes the theme one of the priorities in the area. Therefore, the data can collaborate in health education for older adults, family members and health care staff, including the nurse, professional responsible for providing comprehensive care for the older adult. The importance of identifying extrinsic risk factors for falls in older adults is the possibility of planning strategies for prevention, environmental reorganization and functional rehabilitation. In this context, the objective was to identify the prevalence of falls among older adults and the extrinsic factors associated with them.

## Method

This is a population-based cross-sectional cohort study, addressing long-lived adults living registered in the Family Health Units in the urban area of the city of Foz do Iguaçu, in the state Paraná, conducted from March to June 2015. The participants of this study were older adults aged 80 and over, of both genders, permanent residents in the city. Participants with severe cognitive deficit suggestive of dementia, assessed by the Mini-Mental State Examination and the temporarily or permanently bedridden were excluded. 

The prevalence of falls used for the calculation of the sample, considering an infinite population, was of 30% ^(^ ^16^, with a sampling error of 5% and a significance level of 95%. An additional number of subjects (5%) was included in the minimum sample size as a safety margin, considering possible losses. Data were obtained through a household survey. 

The questionnaire built by the authors was standardized, pre-tested and included socio-demographic variables (age, gender, marital status, level of education, ethnicity and living arrangement), clinical variables (independent walking, locomotion assistance, usage of medication, and presence of diseases) and environmental variables. The dependent variable consists of the existence of a fall event in the year prior to the survey. The questions related to the environment, regarding accessibility, mobility and safety of the older adult were elaborated based on Standard 9050 of the Brazilian Association of Technical Standards. The main places of circulation were observed, such as the main entrance, the living room, the kitchen, the bedroom, the bathroom and the stairs. 

Data were analyzed using Stata Software V.10. Descriptive analyzes included calculations of ratios and the respective 95% confidence intervals. To verify the association between categorical variables, the Pearson’s chi-square test and logistic regression analysis were used with the stepwise criteria for selection of variables in the model, with measures of effect expressed in Prevalence Ratio. Data were analyzed for a significance level of 5%. For input into the multiple model, the variables with p ≤ 0.20 were considered.

The guidelines of Resolution 466/2012 of the National Health Council were respected in the research and all the study participants, along with the researchers, signed two copies of the Consent Form. The study was approved by the Research Ethics Committee of the University of Passo Fundo under protocol number 887.046/2014. 

## Results

The sample of this study consisted of 350 long-lived adults, ten more than the result obtained from the sample calculation, with a mean age of 83.7 years (SD: 3.7 years). The maximum age was 97 years. The majority were female (60.3%), widowed (61.4%), lived with their families (88.9%), lived in households (96.0%), used four or more medications (80.3%) and self-reported as being Caucasians (62.9%). Regarding the level of education, most older adults (74.6%) were illiterate. About 10.0% needed assistance to walk.

The main morbidities presented by the older adults were arterial hypertension (72.9%) and diabetes (25.1%), and the main deficits were visual impairment (70.9%), leg weakness (67.7%), hearing impairment (60.0%) and dizziness/vertigo (59.7%).

Regarding the falls, 46.9% (164) reported suffering a fall in the prior year; of these, 64.4% reported one fall and 35.6% reported two or more. Hospitalization and fear of falling were reported by most of the long-lived adults as the main consequence of the fall, (respectively 34.7% and 34.2%). 31.1% of the cases resulted in fractures. Out of these, 34.3% were of the legs and/or knees, 25.7% of the hip, 24.3% of the shoulders and/or arms and 15.7% of the wrist and/or hand. About 80.0% of the elderly reported falling from their own height, meaning that they fell while walking, and the main cause was slipping (45.1%) and stumbling (26.2%). Most falls occurred inside the house, mainly in the bathroom (26.2%) and in the living room (20.1%).

Regarding the extrinsic factors possibly associated with home falls, the presence of pets (35.1%) and the switch far from the door (32.9%) were observed as main factors, respectively, in the main entrance and in the living room. In the kitchen and in the bedroom, the lack of anti-slip loose throw rugs was observed (respectively 40.9% and 32.3%). In the bathroom, slippery floors were found in 97.7% of the households investigated. In the bivariate analysis, there were statistically significant associations between the occurrence of falls and the variables: age, polypharmacy, Parkinson’s disease, osteoporosis, dizziness/vertigo and perceived health (Table 1) and presence of steps, uneven floor and pets in the main entrance, lack of anti-slip loose throw rugs and slippery floor in the kitchen, lack of anti-slip loose throw rugs and objects in the floor in the bedroom, lack of grab bars in the toilet and in the shower and switch far from the door in the bathroom (p< 0.05) (Table 2). 

**Table 1:** Results of the bivariate analysis of falls and clinical features of the long-lived adults. Foz do Iguaçu, PR, Brazil, 2015 (N=350)

**Variables**		**Falls**			**p**
		**Yes**		**No**	
		**n (%)**		**n (%)**	
**Age**					**0.001**
	**80 - 89 years**	**159 (49.4)**		**163(50.6)**	
	**90 and older**	**5 (17.9)**		**23 (82.1)**	
**Polypharmacy**					**0.003**
	**Yes**	**120 (42.7)**		**161 (57.3)**	
	**No**	**42 (62.7)**		**27 (37.3)**	
**Parkinson’s disease**					**0.001**
	**Yes**	**12 (92.3)**		**1 (7.7)**	
	**No**	**152 (45.1)**		**185 (54.9)**	
**Osteoporosis**					**0.001**
	**Yes**	**18 (72.0)**		**7 (28.0)**	
	**No**	**146 (44.9)**		**179 (55.1)**	
**Dizziness/vertigo**					**0.004**
	**Yes**	**111 (53.1)**		**98 (46.9)**	
	**No**	**53 (37.6)**		**88 (62.4)**	
**Perceived health**					**0.000**
	**Good/Very Good**	**135 (42.5)**		**183 (57.5)**	
	**Fair/Poor**	**29 (90.6)**		**3 (9.4)**	

**Table 2:** Results of the prevalence, crude analysis and adjusted analysis of the extrinsic factors associated to the existing falls in long-live adults’ households. Foz do Iguaçu, PR, Brasil, 2015

**Variable**			**Falls**		PR*	**CI95%**	**PR** ^**†**^	**CI95%**
			**(%)**	**p**				
**Main entrance**								
	**Steps**			**0.000**				
		**No**	**40.5**		**1**		**1**	
		**Yes**	**62.1**		**2.41**	**1.50-3.86**	**1.82**	**1.03-3.21**
	**Uneven floor**			**0.000**				
		**No**	**41.5**		**1**		**1**	
		**Yes**	**84.1**		**7.44**	**3.10-17.89**	**5.54**	**2.26-13.55**
	**Pets**			**0.003**				
		**No**	**41.0**		**1**		**1**	
		**Yes**	**57.7**		**1.97**	**1.25-3.09**	**2.10**	**1.26-3.50**
**Kitchen**								
	**Loose throw rugs**			**0.000**				
		**Yes**	**31.5**		**1**		**1**	
		**No**	**57.5**		**2.94**	**1.85- 4.68**	**3.02**	**1.82-4.99**
	**Tall cabinets**			**0.001**				
		**Yes**	**58.8**		**1.89**	**1.15-3.11**		
		**No**	**43.0**		**1**			
**Bedroom**								
	**Loose throw rugs**			**0.001**				
		**Yes**	**34.5**		**1**		**1**	
		**No**	**52.7**		**2.12**	**1.32-3.94**	**1.84**	**1.08-3.14**
**Bathroom**								
	**Grab bars in the shower**			**0.000**				
		**No**	**42.2**		**1**		**1**	
		**Yes**	**88.6**		**10.61**	**3.66-30.76**	**4.69**	**1.46-15.07**
	**Grab bars in the toilet**			**0.005**				
		**No**	**45.0**		**1**			
		**Sim**	**76.2**		**3.91**	**1.40-10.93**		
	**Switch far from the door**			**0.000**				
		**No**	**44.7**		**1**			
		**Yes**	**88.2**		**9.26**	**2.08-41.14**		

*Crude prevalence ratio

†Adjusted prevalence ratio

In the multiple logistic regression analysis, the following variables remained statistically significant: uneven floor, steps and pets in the main entrance, lack of anti-slip rugs in the kitchen and in the bedroom and lack of grab bars in the bathroom (p < 0.05). The chance of a long-lived adult suffering a fall with the presence of steps in the main access was 1.82 times, with uneven floor of 5.54 times, and with pets of 2.1 times. (Table 2).

The variables tall cabinet in the kitchen, grab bar in the toilet and switch far from the bathroom door lost significance when entering the multiple model.

## Discussion

This is the first population-based, home-based study conducted exclusively with Brazilian older adults aged 80 years or older, investigating the association between falls and extrinsic factors, providing support for identifying risk factors for the prevention of falls. The prevalence of falls was similar to a related study that found a percentage of 43.0% ^(^ ^17^ and is in accordance with the international literature, that presents a percentage of 42.0%^12^. Data from the World Health Organization show that 32.0% to 42.0% of the older adults aged 70 and over suffer falls every year ^(^ ^16^. 

Older ages showed association with a higher number of falls and an increased risk of the event. The biological aging process comprises structural and functional changes that progressively accumulate. These alterations may compromise the performance of motor skills, hinder the individual’s adaptation to the environment and predispose them to suffering falls^8^. Advanced age is closely related to predisposing factors for falls^9^.

Regarding the usage of medication, a higher frequency of falls occurred in the elderly who used four or more medications, which characterizes polypharmacy. The relationship between the usage of medications and occurrence of falls was statistically significant, since the medications may alter motor responses and cognitive capacity, and cause postural hypotension, somnolence, dizziness and the need to urinate more frequently^12^.

Parkinson’s disease and osteoporosis were associated with falls. Parkinson’s disease is a chronic and progressive pathology, characterized by the degeneration of neurons and difficulties in balance. Cognitive decline is indicated as a variable that directly influences the risk of falls in older adults^18^.

Osteoporosis is strongly related to falls, fractures, and declining functional capacity and quality of life. Individuals with osteoporosis may present postural alteration, abnormal gait and body imbalance, which may lead to the occurrence of falls^8^. Most older adults in our study reported dizziness/vertigo. These changes are frequent in older adults and are factors that predispose them to the occurrence of falls ^(^ ^19^. 

Regarding the perceived health status, most older adults who suffered falls reported having good/very good health. However, when considering a fair/poor self-perception, there is a high proportion of falls, about 90%. Perceived health status is as a good indicator of the general health conditions of the elderly population, since it takes into account physical, cognitive and emotional aspects, as well as aspects related to well-being and satisfaction with life. This perception has been widely used in population research with older adults, since it is consistently associated with mortality and functional decline. Besides that, it is also an instrument for the construction of public health policies for this population^20^.

Among those who suffered a fall, one-third had a fracture as a consequence, and most of them (34.3%) were fractures of the legs and/or knees. Studies conducted in the community have shown that fractures are more common in the lower limbs^8^. The higher probability of suffering a fracture as a consequence of a fall is due to the high prevalence of comorbidities in this population^17^. 

The bathroom, the living room and the bedroom were pointed as the places where the elderly fell more frequently. Likewise, a study carried out in the city of Catanduva, in the state of São Paulo, Brazil, with institutionalized older adults found these same places as the main places of falls^21^. 

Extrinsic factors associated with falls are in agreement with the factors found in the literature, such as slippery surfaces, loose throw carpets^22^ ^)^ switches in inadequate places^22^ ^-^ ^23^, narrow or tall steps, obstacles in the way^23^, absence of grab bar in the bathroom, not anti-slip rug in the bathroom and difficult access^23^.

The variables related to the lack of grab bars in the shower, anti-slip loose throw rugs in the kitchen and in the bedroom presented statistically significant association; however, they should be considered with caution. It should be taken into consideration that the research has a cross-sectional design, which, by its nature, does not allow knowing the order of occurrence of the facts. The falls prevalence ratio encountered for the lack of anti-slip rugs might be because the older adults who suffered falls probably removed the rugs from the environment after the event. Also, the same fact might explain the prevalence ratio of falls in the presence of grab bars in the bathroom (in the shower and toilet), which may have been installed as a result of the event, considering that the bathroom was the most frequent place of falls. 

In this study, the cross-sectional design is considered as limitation, since exposure and outcome are collected in a single moment in time, making it difficult to establish a temporal relationship between events and determine whether the relationship between them is causal or not. It is the case for grab bars in the toilet, switch away from the bathroom door and lack of loose throw rugs in the bathroom: the study design does not allow us to identify if those were provided before or after falls. Although the answer to the dependent variable “falls” was obtained through self-report based on recall strategies, it is possible to highlight the possibility of memory bias, since a fall in the last year is an event that will hardly go unnoticed. 

The development of longitudinal studies is suggested, since they might be able of producing new evidences for the prevention of falls and establishment of safety measures for the long-lived adults. The results can also support guidelines to subsidize the construction of public policies and health care programs for this population.

## Conclusion

The study showed a 46.9% prevalence of falls. There was an association between falls and the presence of steps, uneven floor and pets in the main entrance, lack of anti-slip loose throw rugs in the bedroom and in the kitchen and objects on the floor in the bedroom (p<0.05). 

Considering the gravity of the outcomes of falls, it is important that preventive measures are taken by health care professionals, the family and society, in order to maintain independence or minimize impairment of functional capacity and prevent physical damage and hospital admissions, thus, minimizing the high costs that falls bring to the health system and ensuring a good quality of life for this population.
